# Civic Competence of Youth in Europe: Measuring Cross National Variation Through the Creation of a Composite Indicator

**DOI:** 10.1007/s11205-014-0746-z

**Published:** 2014-09-16

**Authors:** Bryony Hoskins, Michaela Saisana, Cynthia M. H. Villalba

**Affiliations:** 1 Department of Social Sciences, Roehampton University, London, UK; 2 Unit of Econometrics and Applied Statistics, Joint Research Centre, European Commission, Ispra, Italy; 3 Department of Education, Institute of International and Comparative Education, Stockholm University, Stockholm, Sweden

**Keywords:** Civic competence, Composite indicator, Active citizenship, Comparative politics, Comparative education, Political participation, Social capital

## Abstract

This article develops a composite indicator to monitor the levels of civic competence of young people in Europe using the IEA ICCS 2009 study. The measurement model combines the traditions in Europe of liberal, civic republican and critical/cosmopolitan models of citizenship. The results indicate that social justice values and citizenship knowledge and skills of students are facilitated within the Nordic system that combines a stable democracy and economic prosperity with a democratically based education systems in which teachers prioritise promoting autonomous critical thinking in citizenship education. In contrast, medium term democracies with civic republican tradition, such as Italy and Greece gain more positive results on citizenship values and participatory attitudes. This is also the case for some recent former communist countries that retain ethnic notions of citizenship. In a final step we go on to argue that the Nordic teachers’ priority on developing critical and autonomous citizens perhaps facilitates 14 years olds qualities of cognition on citizenship and the values of equality but may not be the most fruitful approach to enhance participatory attitudes or concepts of a good citizen which may be better supported by the Italian teachers’ priority on civic responsibility.

## Introduction

Cross-country comparisons of young people’s qualities for civic engagement have become an established field of research inquiry (Hahn 1998; Torney-Purta et al. 1999, 2001; Amnå and Zetterberg 2010). In the European Union member states, recently undergoing economic crisis and a reduction of global power, it can no longer be taken for granted that the region will remain stable and democratic (Europe’s Political Union 2012). Therefore, there is a growing need to monitor the learning outcomes of democracy through comparative research. With appropriate indicators, country differences and changes across time in young people’s knowledge, attitudes and values, and intended behaviour can be monitored. In this context, this article develops four dimensions of youth civic competence using International Civic and Citizenship education Study (ICCS) data (Schulz et al. 2010) to examine the levels of civic competence in young people aged about 14 years across Europe. It aims to contribute to the debate on how to measure and monitor the complex notions of citizenship and the learning of citizenship. Going one step further, the article will explore why young people in different countries might differ in their levels of civic competence, drawing on comparative political theory (Almond and Verba 1963; van Deth et al. 2007; Kohn 2008) and theories of Citizenship Education (Hahn 1998; Torney-Purta 2002).

The youth civic competence measure presented in this paper, civic competence composite indicator-2 (CCCI-2), is based upon the pilot version developed using the IEA CIVED data (1999) (Hoskins et al. 2011). However, considerable changes have been made in the new version of the ICCS study instruments, making it difficult to draw direct comparisons (Barber and Torney-Purta 2012). Accordingly, we have taken the opportunity to improve the measurement instruments rather than focus on assuring comparability across the two datasets. Nevertheless, we have been able to maintain the composite indicator’s structure and basic constructs of the four dimensions of citizenship values; participatory attitudes; social justice; and a cognitive dimension that we have renamed ‘Knowledge and Skills for Democracy’.

The first section of the article defines civic competence using the concepts of liberal, civic republican and critical/cosmopolitan citizenship. Section 2 identifies theories that help us to understand cross-national variation in the context of democratic stability and citizenship education among countries selected for analysis. Section 3 explains the data and describes the methodology for creating the composite indicator of civic competence and its four dimensions. Section 4 presents the results and reflects on the extent to which current theories help to provide useful explanations. Sections 5 and 6 conclude the article by identifying the study’s limitations, which provide direction for future research and insight on how policy, and thus society, can benefit from the use of complex composite indicators by shedding light on key social issues such as the preparation of young citizens for future active citizenship.

## Defining Civic Competence

This section defines civic competence by drawing on the three dominant concepts of citizenship that have influenced its construction across Europe: liberal, civic republican and critical/cosmopolitan citizenship (Hoskins and Kerr 2012). We will visit each of these to investigate how they have shaped its understanding, hypothesizing that the political history of a country in terms of its concept of citizenship will have influenced the development of different facets of civic competence. In other words, as we will explain in Sects. 1–3 below, the dominant political paradigm of a country interact with other factors to form a theoretical context for interpreting the results of the quantitative analysis.

### The Liberal Concept of Citizenship

During the period 2006–2009 (before data collection was completed), the vast majority of elections in the European Union brought power predominantly to centre-right parties, influencing the dominant liberal model of citizenship across Europe (Hoskins and Kerr 2012). There is a long history of a liberal citizenship concept in the Anglo-Saxon countries of Europe. In its original meaning, liberal democracy is typically considered ‘thin’ democracy. This means that citizens’ involvement in public life is minimal, and is primarily enacted through the vote (Delli Carpini and Keeter 1996). In such an environment, citizens are encouraged but not obliged to vote. Education for active citizenship is focused on creating autonomous citizens who can act to support their own self-interest and to enhance individuals’ basic level of political knowledge and skills to achieve this end. Active citizenship under the liberal concept emphasizes the rights of individuals to participate (or not) politically. The implications of the liberal approach on civic competences have been to focus on knowledge, skills and dispositions towards engagement. However, Honohan (2002) asserts that without civic virtues, an excess of the self-interest associated with the liberal model can lead to corruption.

### The Civic Republican Concept of Citizenship

Many European countries have civic republican roots, whether due to influence by France and the narrative of the French Revolution (including much of southern Europe) or from a legacy of civic concepts of nationalism, such as in Greece and Italy (Kohn 2008). Civic republicanism has also been associated with nationalist policies developed more recently in many former communist countries after independence (Toots and Idnurm 2011), although these tend to have a more ethnic than civic concept of citizenship (Kohn 2008). The civic republican approach places higher demands on the citizen in terms of maintenance of the democratic processes and institutions, which in turn assure greater freedoms. From this perspective, citizens become the actors of positive laws for social change and are the instruments to prevent corruption (Lovett 2010). Civic republicanism emphasizes the need for citizens to act politically within the public sphere, in particular at the national level, and to be actively engaged within a political community as equal and free citizens. Thus, the notion of civic responsibility has developed from this view.

Compared to the liberal tradition, this approach assigns both a greater obligation and greater value to political engagement and involvement in political decision making. In terms of civic competence, the qualities of knowledge, skills, values and attitudes to enable political engagement are of the highest importance. They are, for example, the qualities needed to evaluate the performance of government, the skills to recognize and prevent corruption, and the dispositions and abilities to participate in public discourse (Galston 2001). The civic republican approach also highlights the need for citizens to learn civic virtues, and stresses the value of public spiritedness, solidarity and responsibility to act for the common good (Honohan 2002: 147). It is necessary to acknowledge that civic republicanism is also associated with the values of patriotism and nationalism that have been widely criticised for undermining that of equality for immigrants and minorities, and for a disregard for human rights in the process of achieving the *common good* (Abowitz and Harnish 2006).

### The Critical/Cosmopolitan Concept of Citizenship

In recent years, the governments of European Union countries have not accorded the highest policy emphasis to critical/cosmopolitan citizenship (Hoskins and Kerr 2012). Nevertheless, the values of equality and human rights featured in this model have a considerable history in Nordic countries, both within and beyond the educational system where social rights and economic redistribution are traditionally supported (Telhaug et al. 2006). The critical citizenship model has been a ‘catch-all’ title for various new theories that try to frame active citizenship in different terms (Abowitz and Harnish 2006), for example by focusing on critiquing and improving equality in society through social and political action (Johnson and Morris 2010). Aspects of civic competence considered prerequisites for critical citizenship are the ability critically to analyse ‘social issues and injustices’, for example learning to ask why people are homeless instead of merely collecting money to feed them (Westheimer and Kahne 2004: 4) and other social values such as empathy and care (Veugelers 2011). The concept of cosmopolitanism, one form of the critical citizenship concept, aims to move beyond national citizenship to a global concept of humanity with internationally recognized human rights and the valorization of diversity (Held 2010).

### Civic Competence Model

**Fig. 1:**
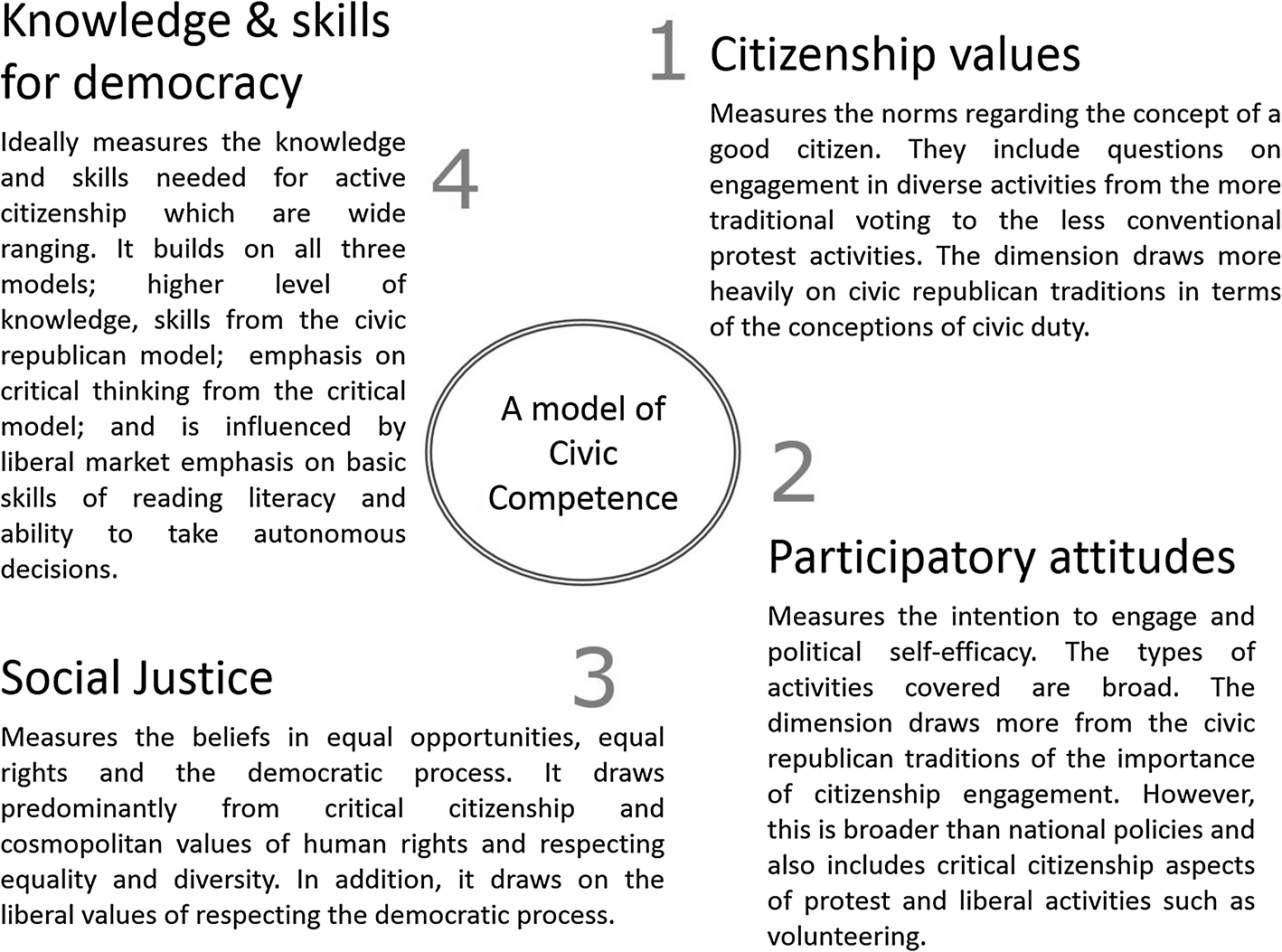
A model of civic competence used to develop CCCI-2 and CCCI

The pilot framework for youth civic competence was developed as part of the ‘Active Citizenship for Democracy’ study (Author) by experts across Europe. It draws elements from citizenship concepts described above but not explicit in the original instrument (Fig. 1). The citizenship values dimension incorporates the norms of a good citizen and draws substantially on the civic republican discourse of civic duty. The participatory attitudes dimension measures disposition to engage, again drawing on civic republican ideals of participation. The social justice dimension measures cosmopolitan values of human rights and respecting diversity. It also encapsulates liberal attitudes of respect for the democratic process. The Knowledge and Skills for Democracy dimension transcends all three models, and measures the full range of skills needed to be an active citizen.

## Factors Influencing Cross-National Variation

In this section we explore two possible factors behind cross-national variations in youth civic competences: length of stable democracy and citizenship education. A priori, we would expect wide variation in youth civic competence due to the diverse political and cultural histories and education policies on citizenship in the countries.

### Length of Stable Democracy

A measure frequently employed by comparative political science of democratic culture is the duration of stable democracy (Almond and Verba 1963: van Deth et al. 2007). The theory is that, within stable democracies, the experience of political involvement in democracy is passed down from one generation to the next through political socialisation (Almond and Verba 1963). In direct contrast to findings for the adult population (van Deth et al. 2007), a negative association was found between longer periods of democracy and youth participatory attitudes and citizenship values, using the first composite indicator (Author). Thus, young people from countries experiencing a relatively recent transition to democracy, therefore with less political stability, appeared more highly to value democratic participation. It has thus been argued that this greater intention to participate is due to the very fragility of the democratic institutions, and that within this age group the instability of political external factors and recent memory of an absence of democracy have generated the values associated with civic competences (Torney-Purta et al. 2008). It is interesting to note from Almond and Verba’s (1963) seminal text on the influence of stable democracy on civic culture that people in more recent democracies often idealise political participation, as they have little actual experience to draw from. Thus, what may be depicted by the young people in more recent democracies is a more idealised vision of democracy and their potential involvement than that in older democracies. If stability of democracy appears to have a negative impact on citizenship values and participatory attitudes, then the following country groupings are what we would expect to see:

1. Former communist countries. These countries have experienced communist regimes and a very recent transition to liberal democracy (e.g. Bulgaria, Czech Republic, Estonia, Latvia, Lithuania, Poland, Slovak Republic, Slovenia);
2. Countries that experienced breaks in democracy and have undergone fascism, dictatorship, and/or occupation and then only recently (within the past 65 years) a transition back to democracy (e.g. Greece and Cyprus), as well as countries that have experienced fascism and rather unstable transitions to democracy after the Second World War (e.g. Italy);
3. Countries that have had a stable and continuous experience of democracy over the past 65 years or more (e.g. Sweden, Norway, Denmark, Finland and England). However, it should be noted that some of these countries, such as Norway and Denmark, were occupied by Nazi Germany during the Second World War, moreover Finland was invaded by the Soviet Union during this period and was eventually forced to cede a part of its territory to the Soviets in order to maintain its sovereignty.

This hypothesis for the impact of democracy on youth civic competence was proved accurate in describing the results from the pilot composite indicator for the dimensions of participatory attitudes and citizenship values; the expectation is that we shall find similar results again.

### Citizenship Education

Considerable research has been undertaken to identify how the various forms of citizenship outcomes are learnt by young people, both within and outside school, in the community and at home. In this way, ‘citizenship education’ is more complex and harder to demarcate in terms of content, organization and implementation strategy than a typical school subject such as maths. Moreover, citizenship learning is often reinforced through informal channels available to young people in different settings. In this section we explore the literature on informal ways of learning citizenship as well as that on citizenship education.

One key finding has been that diverse social and civic forms of participation have been associated with the learning of civic knowledge, skills and attitudes. Kahne and Sporte (2008) in the US found that all experiences that focus directly on civics and political issues impact upon the commitment to community participation. In particular, they highlighted that the most significant results related to learning through volunteering (service learning). They also noted that extra-curricular activities, exposure to civic role models and open debates were predictors of intention to participate in the community. A positive impact by volunteering on participation levels in later life was also found in other US studies (Verba et al. 1995; Campbell 2006).

At school, social and civic forms of participation have also been associated with learning civic competence. An open classroom climate has consistently been shown to be positively associated with higher levels of civic competence (Hahn 1998; Hoskins et al. 2012; Torney-Purta 2002), and school councils and parliaments have also been shown to have positive effects (Torney-Purta 2002). The democratic ethos of the whole school has also been identified as a key factor in enhancing an individual’s self-efficacy and willingness to participate (Benton et al. 2008). Democracy is said to pervade the Nordic education system, from school ethos to classroom practice (Telhaug et al. 2006), and one would expect countries in this region to have high levels of all aspects of civic competence. Nevertheless, Janmaat (2013) has suggested that there may well be different approaches needed to form different aspects of civic competence. He suggests that effective methods for promoting participatory attitudes may well be quite different from those promoting tolerance. As we visit the results for each facet of civic competence, we will explore the teaching or curricular approaches dominant in the countries where young people have high levels of the various aspects of civic competence.

There are three main Citizenship education strategies: discrete lessons, integration into other disciplines, and a cross-curricular approach (Eurydice 2012). The evidence for the positive effects of discrete lessons or cross-curricular format paints a mixed picture in terms of positive associations with civic competences. One of the most in-depth and up-to-date research projects on citizenship education is England’s Citizenship Education Longitudinal Study (CELS) (2001–2010). The CELS researchers found only limited evidence of any positive association between citizenship education, whether organised through a cross-curricular approach or specific individual lessons, and the outcomes of civic knowledge and participatory intention (Benton et al. 2008; Sturman et al. 2012). The Citizens in Transition study, a round added to the CELS study in 2011 following a UK general election, found more positive results. For example, Whiteley (2012) found citizenship education had an effect on efficacy, civic knowledge and political participation. However, it should be borne in mind that this association was for a complex mix of subjective and objective information, combining a self-reported measure of the citizenship education received, along with the specific national curriculum the student followed (students in England underwent compulsory citizenship education as either discrete lessons or cross-curricular, whereas students in Scotland and Wales had it integrated into other disciplines). In contrast, research based on the IEA CIVED data for Finland, Germany, Poland, Italy and England found no positive association between an objective measure of the hours spent studying social science subjects, including citizenship education, and levels of citizenship knowledge, skills or participatory attitudes (Hoskins 2011). Nevertheless, Niemi and Junn (1998) found that civics courses in the US helped to develop civic knowledge and skills, a finding supported by evaluation research on citizenship education programmes in Poland (Slomczynski and Shabad 1998), Bosnia (Soule 2000) and post-apartheid South Africa (Finkel and Ernst 2005).

Citizenship education is part of the compulsory curriculum of all the countries investigated and, according to Eurydice (2012), all students experienced some citizenship education prior to participation in the ICCS study. Eurydice (2012) completed the most comprehensive and recent comparison of citizenship education across European countries and notes that the main differences lie in the strategies for the implementation of citizenship education, that is, whether delivered in discrete lessons, integrated into other disciplines or through a cross-curricular approach. The difficulty in making comparisons arises from the fact that different strategies may be applied within a single country by different schools for the same academic year. In addition, there has been a significant number of curriculum reforms on citizenship education during the last 5 years (Czech Republic in 2007; Latvia in 2006; Lithuania in 2009; Finland in 2004; Norway in 2006; Italy in 2008), which adds an element of uncertainty regarding the precise nature of the implementation of the surveyed students’ citizenship education, thus a clear link between citizenship education and our results is difficult to identify. Nevertheless, when countries perform differently from others with similar socio-political histories, an examination of their citizenship education is worthwhile. It is also the case that in Europe it is the implementation, rather than the policies and the curriculum, that has been cited as the main culprit behind lower citizenship outcomes (Bîrzéa et al. 2004). Taking this on board, we also consider the perspectives of teachers on the objectives of citizenship education, since this indicator may be a proxy for the actual experience of students.

Eurydice performed an analysis on teachers’ perceptions of civic and citizenship education using ICCS 2009 data on teachers in the 21 European countries that participated in this part of the study. Responses were analysed to the question that asked teachers to select the main aims of citizenship education from a list of ten possibilities. There was a wide degree of country variation: almost 80 % of Italian teachers chose promoting knowledge of citizens’ rights and responsibilities, in contrast to Austrian teachers of whom <20 % identified this as a priority. Eurydice (2012) found that in the Nordic countries (Denmark, Sweden and Finland), over 80 % of teachers prioritized promoting students’ critical and independent thinking, in contrast to many Eastern European countries (Czech Republic, Poland and Slovakia), where less than half gave priority to this issue. Thus, we could expect that countries such as Italy, where teachers prioritized rights and responsibilities of students, would perform higher on citizenship values and participatory attitudes, whilst countries with teachers who prioritized critical and independent thinking would perform higher on the citizenship knowledge and Skills tests.

## Measuring Civic Competence Using the IEA ICCS Study

### Data Source, Sample Design and Scales

In this section we describe the main data source used, the IEA ICCS study; how the sample for each country was created; and the scales that we have used in our analysis.

ICCS is the most recent IEA study on civic and citizenship education among school pupils, conducted in Europe in 2009. It was a school-based study that included over 140,000 students, 62,000 teachers and 5,300 school principals from 38 countries. The ICCS student population comprised students from Grade 8 (pupils of approximately 14 years of age, although some were above and below this age) (Schulz et al. 2010), provided that the average age of students in this grade was 13.5 years or above. If the average age of students in Grade 8 was below 13.5 years, Grade 9 became the target population. Anonymity was maintained for all participating students and schools. National coordinators were responsible for following additional national ethical procedures for their countries.

All participating countries were expected to define the population of relevant schools, then the population of the classes and students in these schools (including information on age and gender) according to the IEA guidelines (Schulz et al. 2011). Some countries excluded schools in remote regions or segments of their educational system in order to limit coverage according to national restrictions, for example if a school’s curriculum differed from the mainstream. Non-native language schools (in respect of language of the test) were also excluded. In the same way, there are several types of populations not considered, for example schools and students in remote areas, or students with severe disabilities. The IEA recommended that <5 % of the national population should be excluded from the study (Schulz et al. 2011). The main limitation of this construction of the population is that it reflects only mainstream education; students’, whose voices are already marginalised, due to disability for example, are excluded still further by being unable to participate.

The IEA Data Processing Centre (DPC) was responsible for the sampling process and implemented a two-stage sampling design (Schulz et al. 2011). In the first step the schools were sampled within each country using the technique of probability proportional to size, measured by the number of students enrolled in a school (Schulz et al. 2011). The IEA recommended that a minimum of 150 schools per country should be selected to achieve the appropriate level of precision. In the second step, one class per school was selected randomly by the DPC and all students in that class participated. In small countries such as Cyprus, two classes were sampled.

The national coordinators were responsible for conducting the national studies according to IEA guidelines and compiling the national datasets. These were then sent to the DPC to be checked, cleaned and combined into an international dataset.

Quality control for this study was organised by the IEA Secretariat and national representatives nominated by each country. These representatives were responsible for observing and reporting on implementation of the study, and reviewing the translation verification procedure. For more details about how the IEA ICCS study was conducted, see Schulz et al. (2011).

The study covered four themes:

- civic society and systems;
- civic principles;
- civic participation; and
- civic identities.

Several data collection instruments were administered in each participating country, two of which were used in the construction of CCCI-2:

- An international cognitive student test consisting of 80 items measuring civic and citizenship knowledge, analysis, and reasoning. The items were assigned to seven booklets according to a balanced, rotated design. Each student completed one of the 45-min booklets.
- A student questionnaire consisting of items measuring student background variables and students’ attitudes and behaviours.

The cognitive items were typically presented as units in which some brief contextual stimulus (an image or some text) was followed by relevant questions. Seventy-three items were multiple choice and six required a constructed response (Schulz et al. 2010: 59). The affective-behavioural aspects included questions on *value beliefs*, *attitudes*, *behavioural intentions* and *behaviour* and these were measured using the student questionnaire (Schulz et al. 2010: 26). In most cases the response categories were a set of Likert-type items of four categories (e.g. ‘strongly agree,’ ‘agree,’ ‘disagree,’ and ‘strongly disagree’). The items were then recoded so that higher scores corresponded to more positive attitudes.

In this article we analyse the results for the 16 countries of the European Union and/or European Economic Area that participated in both the 2009 ICCS and the 1999 CIVED study. These countries are: Bulgaria, Cyprus, Czech Republic, Denmark, England, Estonia, Finland, Greece, Italy, Latvia, Lithuania, Norway, Poland, Slovak Republic, Slovenia and Sweden.

#### Scales

The scales we used for the CCCI-2 were all developed by the IEA. Responses to individual items on the questionnaire were combined to create scales that provided a more comprehensive view of the intended construct than could single individual variables (Brese et al. 2011). They were normally calculated as ‘IRT WLE scores with a mean of 50 and a standard deviation of 10 for equally weighted countries’ (Brese et al. 2011: 20) and based on the responses from the full dataset, including 140,650 students in 38 countries (2009 IEA ICCS survey).

### Operationalizing Civic Competence: A Framework

In this section we explain how we operationalized civic competence in the context of the data available in the 2009 IEA ICCS dataset, and built the CCCI. The overall strategy for creating the CCCI-2 was to adopt the same theoretical foundations and concepts from the pilot index, then to select the same or similar scales as previously and to integrate new measures where needed. In this way the knowledge and experience gained during construction of the pilot index could be used to build CCCI-2.

**Fig. 2:**
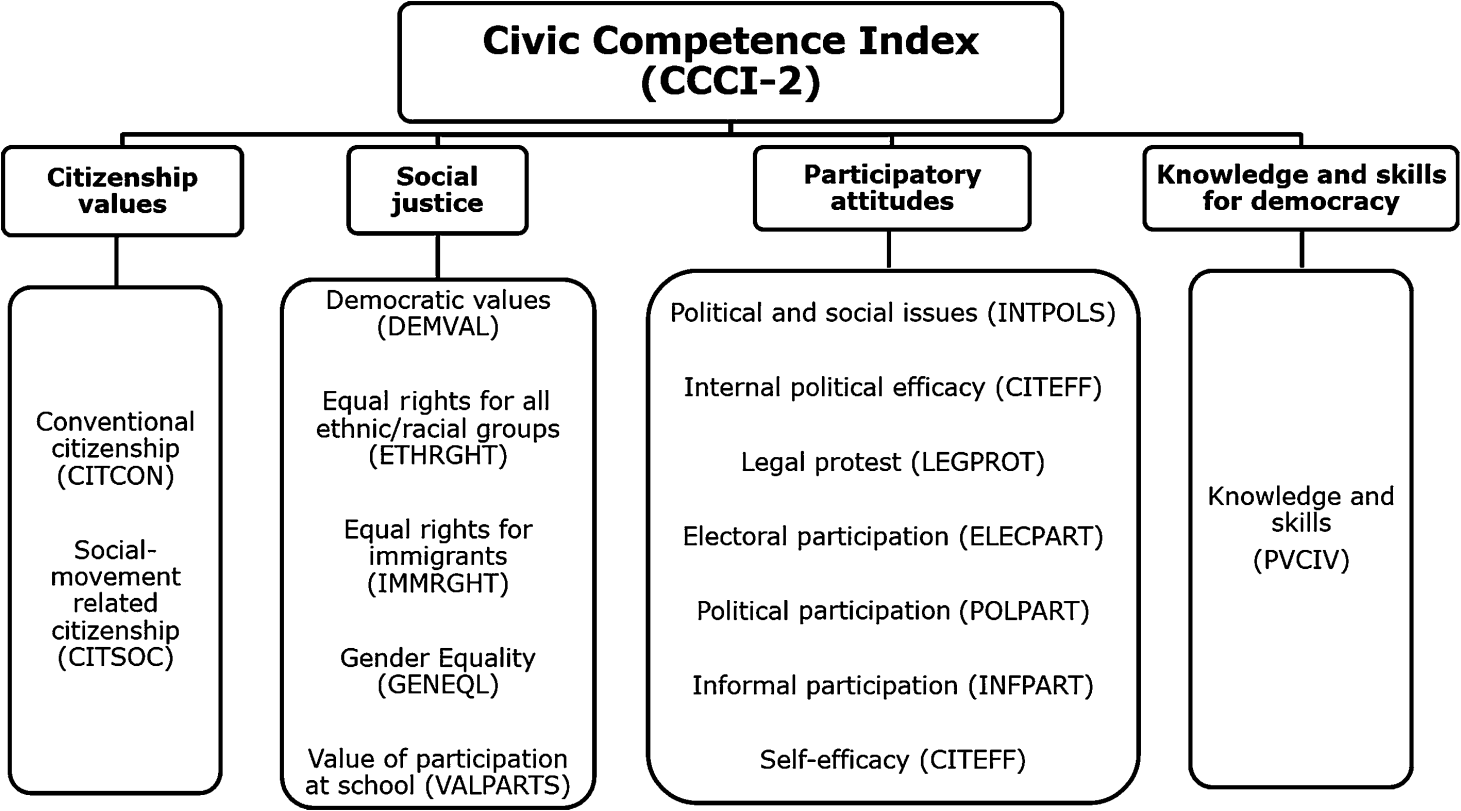
The conceptual framework of civic competence

The conceptual framework of CCCI-2 builds on four dimensions: citizenship values, participatory attitudes, social justice and Knowledge and Skills for Democracy (Fig. 2). The first dimension, citizenship values, has two scales: norms of conventional citizenship, and norms of social movement related citizenship (CITCON, CITSOC). The participatory attitudes dimension includes measures of political self-efficacy (CITEFF), expected participation in political activities (POLPART), expected adult electoral participation (ELECTPART), expected adult informal political participation (INFPART), expected legal protest (LEGPROT), and interest in political and social issues (INTPOLS). The social justice dimension includes measures of democratic rights (DEMVAL), equal rights for ethnic groups (ETHRIGHT), equal rights for immigrants (IMMRGHT), Gender Equality (GENEQUL) and valuing democratic processes at school (VALPART). The dimension on Knowledge and Skills for Democracy contained the cognitive score from the ICCS test.

**Fig. 3:**
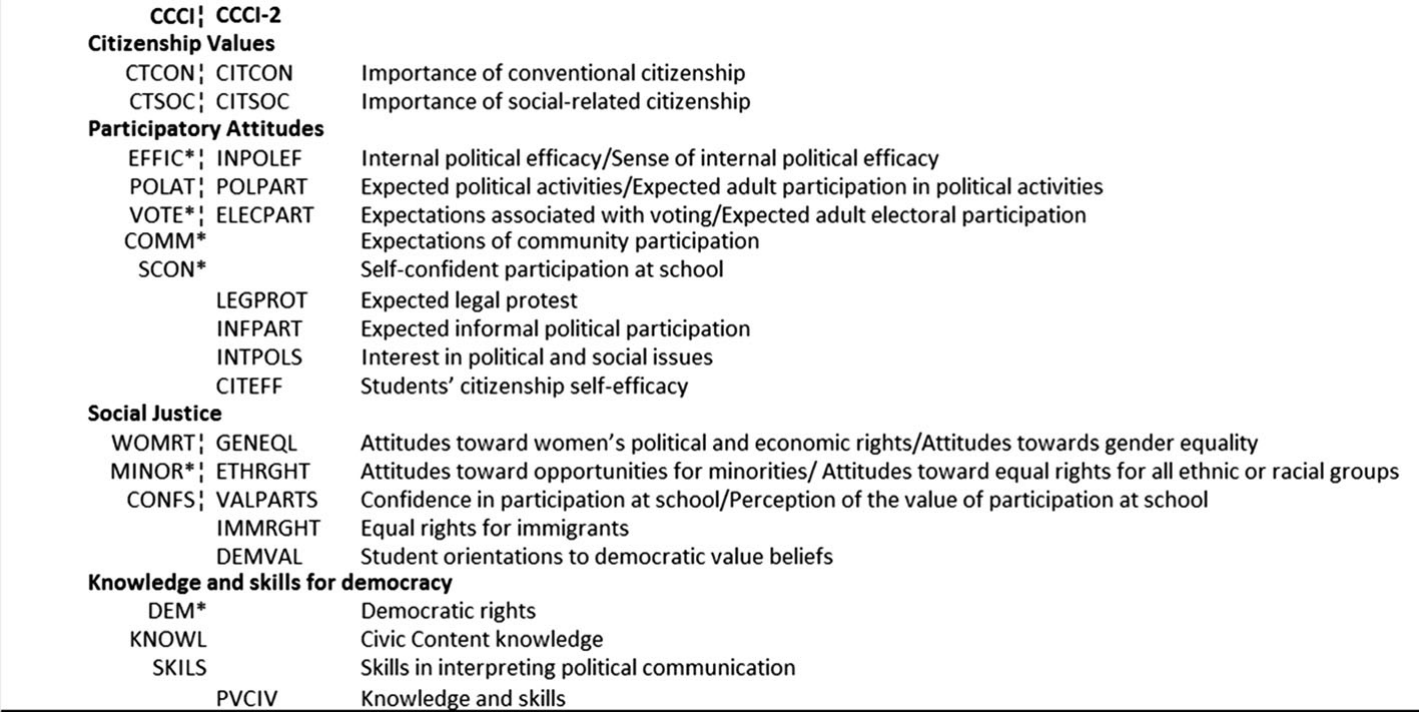
A comparison between the civic competence scales used in CCCI and CCCI-2. *Note* *non-IEA developed scales in the CCCI. All CCCI-2 scales were developed by the IEA. The CCCI scales are based on survey data from the CIVED (1999). The CCCI-2 scales are based on survey data from the ICCS (2009)

Hence, the CCCI-2 model is built from 15 scales; eight scales are similar to those used in the pilot index (on 1999 data) and six are either entirely new or have been moved to a new dimension (Fig. 3). Finally, the Knowledge and Skills dimension is measured by a new cognitive scale, with only a small number of common items (PVCIV). In addition, there are some differences between the last two IEA citizenship studies including changes in the response items and IRT scaling (Barber and Torney-Purta 2012). Therefore, the CCCI-2 is not directly comparable to the pilot index. Nevertheless, there are some similarities.

To create the measurement model on youth civic competence, we followed the methodological guidelines of the *Handbook on Constructing Composite Indicators* (OECD/EC JRC 2008) that we describe next.

### Creating the Civic Competence Indicator

The theoretical framework was based on selected literature and results of the pilot index mentioned previously (Author). The next step was to identify the extent to which the framework had statistical support from current ICCS data.

The assessment of the statistical coherence of the framework was undertaken by first applying principal component analysis (PCA) to the dataset to identify the main ‘statistical’ dimensions of civic competence, then applying factor analysis (FA) to analyse the ‘statistical’ grouping of the scales. These analyses were applied to the full dataset of all 38 countries participating in the 2009 IEA ICCS survey (more than 140,000 students). The PCA identified four ‘statistical’ dimensions (with eigenvalues >1.0), which together explain more than 60 % of the total variance in the 15 scales. FA was applied to extract four principal factors after an orthogonal rotation with Kaiser Normalization (Kaiser 1960).

Table 1 presents the loadings (correlation coefficients) of the indicators with each of the four factors. Numbers in bold reflect the highest factor loading of a scale, and numbers in italics are considered sufficiently high to be taken into consideration when interpreting the results.

**Table 1:** Statistical grouping of indicators into dimensions of civic competence

Civic competence dimension	Civic competence indicator	Factor			
		1	2	3	4
Citizenship values	Conventional citizenship	.303	.124	**.760**	−.137
	Social-movement related citizenship	.120	.372	**.700**	−.009
Social justice	Democratic values	.007	*.489*	.271	.388
	Equal rights for all ethnic/racial groups	.136	**.759**	.158	.179
	Equal rights for immigrants	.102	**.816**	.060	.020
	Gender equality	−.043	*.440*	−.096	**.642**
	Value of participation at school	.169	*.444*	.402	.185
Participatory attitudes	Political and social issues	**.568**	−.088	.500	.077
	Internal political efficacy	**.653**	−.104	.385	.166
	Legal protest	**.714**	.277	−.018	.017
	Electoral participation	**.553**	.105	.283	.355
	Political participation	**.751**	.017	.073	−.240
	Informal participation	**.806**	.100	.135	−.119
	Self-efficacy	**.678**	.152	.187	.065
Knowledge and skills for democracy	Knowledge and skills	−.022	.069	−.026	**.885**

Maximum likelihood Factor loadings (correlation coefficients) obtained after orthogonal rotation with Kaiser normalization

The FA results confirm the conceptual framework for measuring civic competence. The first factor captures ‘participatory attitudes’ (all seven scales), as conceptualized. The second summarizes ‘social justice’ (all five scales), and the third comprises the two scales on ‘citizenship values’. Finally, the fourth factor describes mostly ‘Knowledge and Skills for Democracy’. The Gender Equality scale co-varies between the ‘Knowledge and Skills’ dimension and the ‘social justice’ dimension. For theoretical reasons, we believe that it is more meaningful to place Gender Equality within the social justice dimension rather than the Cognitive dimension as it forms a belief scale of issues of equality rather than cognitive quality. The reliabilities (internal consistencies among the scales measured by the Cronbach’s alpha coefficient) of the four dimensions of civic competence are also adequate, varying from .67 (citizenship values) to .84 (participatory attitudes).

Besides the confirmation on the conceptual grouping of scales into four dimensions, FA results offer a further suggestion that stems from a consideration of the factor coefficients and factor loadings. The coefficients and the loadings of the scales within ‘citizenship values’ and ‘participatory attitudes’ are positive and of the same magnitude, which suggests that building the respective dimension as a simple average of the underlying scales is statistically supported by the data. By contrast, the ‘social justice’ dimension is mostly determined by *equal rights for all ethnic/racial groups* and *equal rights for immigrants* (notice the almost double loading of those two scales compared to those of the other three). To this end, in order to arrive at a balanced dimension on ‘social justice’ where all five scales have similar contributions, the two scales on equal rights are further combined into a single scale by taking their average. This is because the scales on ethnic/racial groups and immigrants measure similar constructs, and to avoid double counting it makes sense to combine the two.

The 15 scales populating the CCCI-2 civic competence framework were not set to the same international mean and standard deviation; furthermore, some of the scales had different units of measurement. To render the 15 scales comparable, different normalization techniques can be used (see OECD/EC JRC 2008). The most common approach is the Min–Max, which was used in the previous version of the CCCI and is used here. The normalized score for an individual in a given scale is given by

1 $$ I_{icj} = \frac{{x_{icj} - min_{cj} (x_{icj} )}}{{max_{cj} \left( {x_{icj} } \right) - min_{cj} (x_{icj} )}} $$

where the subscript *i* refers to an individual (student), *c* refers to the country and *j* to the scale. After this normalization step, all 15 scales range between 0 (lowest score) and 1 (highest score) at the individual level.

The CCCI-2 civic competence composite indicator is built using a simple arithmetic average across the scales within each of the four dimensions, then a simple arithmetic average across the four dimensions. Thus, the civic competence composite indicator CCCI-2 score for an individual is given by the simple average of the scores obtained in each of the four dimensions, that is

2 $$ Y_{cj} = \frac{1}{4}\mathop \sum \limits_{i = 1}^{4} D_{icj} $$

The dimension score for an individual is the weighted average of the normalized scales underlying a given dimension, namely

3 $$ D_{cj} = \frac{1}{k}\mathop \sum \limits_{i = 1}^{k} I_{icj} $$

All normalized scales receive equal weights within a given dimension (example: conventional citizenship and social movement related citizenship receive half weight in the dimension ‘citizenship values’). The only exceptions are *Equal rights for all ethnic/racial groups* and *Equal rights for immigrants* indicators in the ‘social justice’ dimension, which receive one-eighth weight each, while the others receive a quarter.

The CCCI-2 score at national level, *Y*_*c*_, for a given country *c,* is the average CCCI-2 score across the country’s individuals

4 $$ \overline{{Y_{c} }} = \frac{1}{N}\mathop \sum \limits_{j = 1}^{N} Y_{cj} $$

and the corresponding standard deviation is

5 $$ SD_{c} = \sqrt {\frac{{\mathop \sum \nolimits_{j = 1}^{N} (\overline{{Y_{c} }} - Y_{cj} )^{2} }}{N - 1}} $$

A robustness analysis was also conducted to examine how the results, at country level, are affected by changing assumptions about two sources of uncertainty (the normalization process and the structure of the composite indicator, using the framework based on the FA results for each single country). The results of the robustness analysis showed no major differences from the results that we present here (Author).

#### Relationship Between the Four Dimensions of Civic Competence

Table 2 presents the Pearson correlation coefficients between the four dimensions of civic competence at the individual level. The highest association was observed between ‘citizenship values’ and ‘participatory attitudes’, with a correlation of .49. The scores for ‘social justice’ show a moderate link with both ‘participatory attitudes’ (r = .26) and the cognitive dimension (‘Knowledge and Skills for Democracy’, r = .4). However, there is no relationship between cognition, on the one hand, and either ‘citizenship values’ or ‘participatory attitudes’ on the other. Overall, these moderate to practically random correlations suggest that the four dimensions capture distinct aspects of civic competence with barely any or no overlap of information.

**Table 2:** Pearson correlations between the four dimensions of civic competence

	Social justice	Participatory attitudes	Knowledge and skills for democracy
Citizenship values	.37***	.49***	−.01
Social justice		.26***	.4***
Participatory attitudes			.02

*** Significant correlation coefficients at 99 % level (n ≈ 140,000)

## Results

**Fig. 4:**
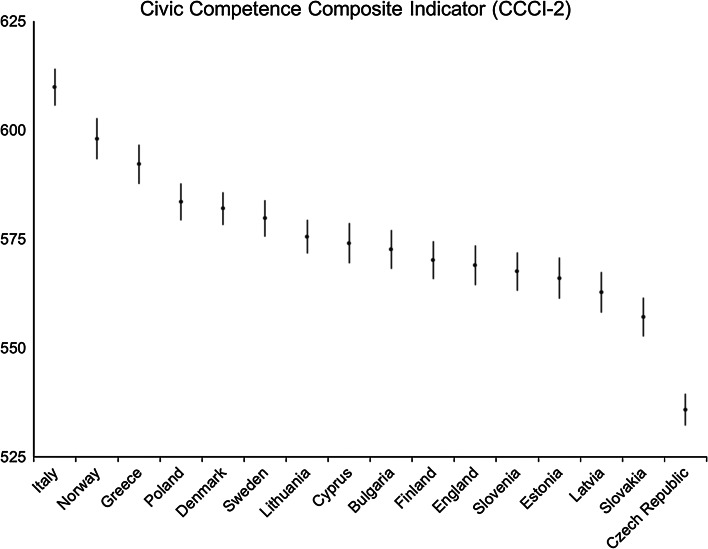
Civic competence: average country scores (with confidence intervals). *Note* confidence intervals are calculated at 95 % level based on a multi-comparison test

**Fig. 5:**
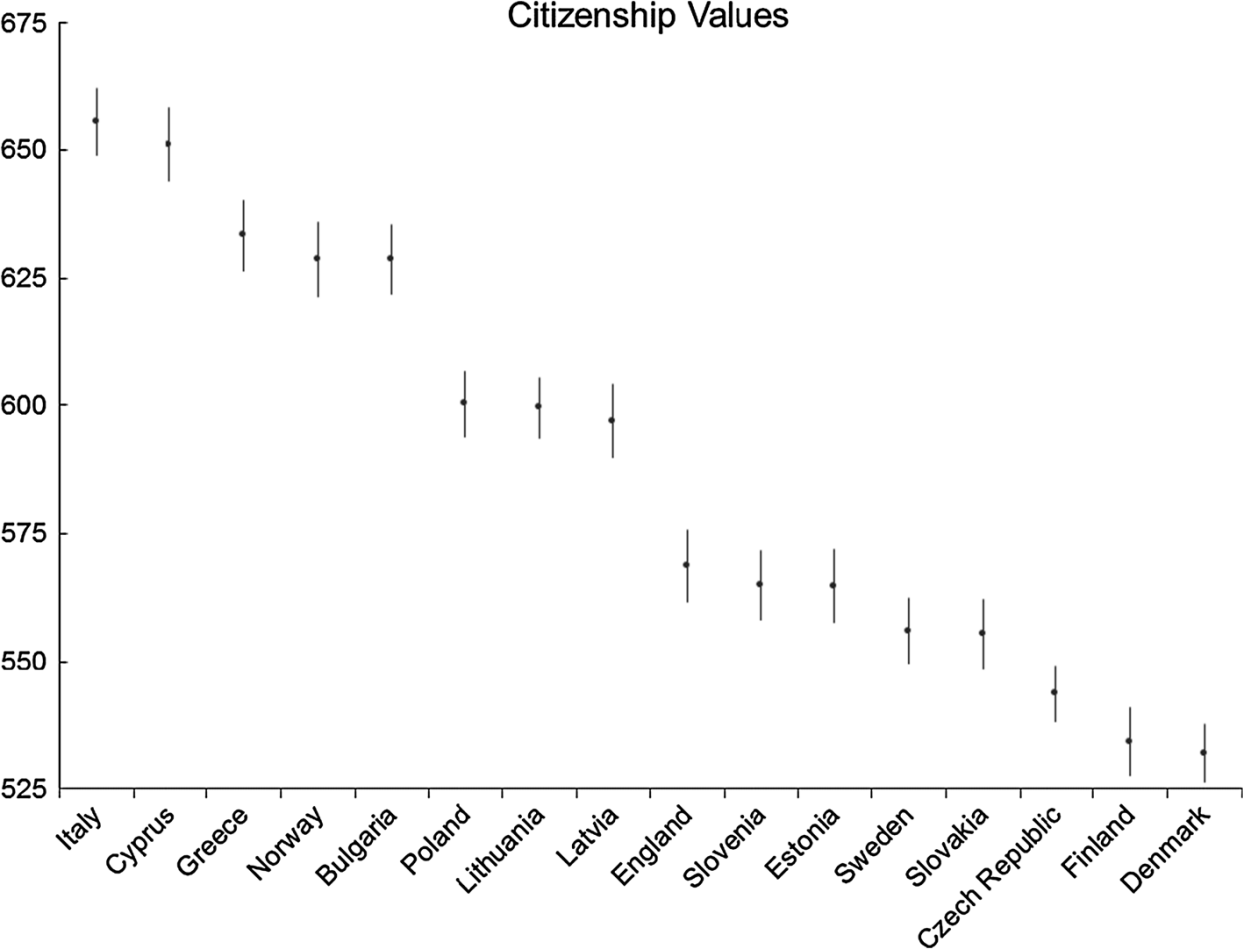
Citizenship values: average country scores (with confidence intervals). *Note* confidence intervals are calculated at 95 % level based on a multi-comparison test

**Fig. 6:**
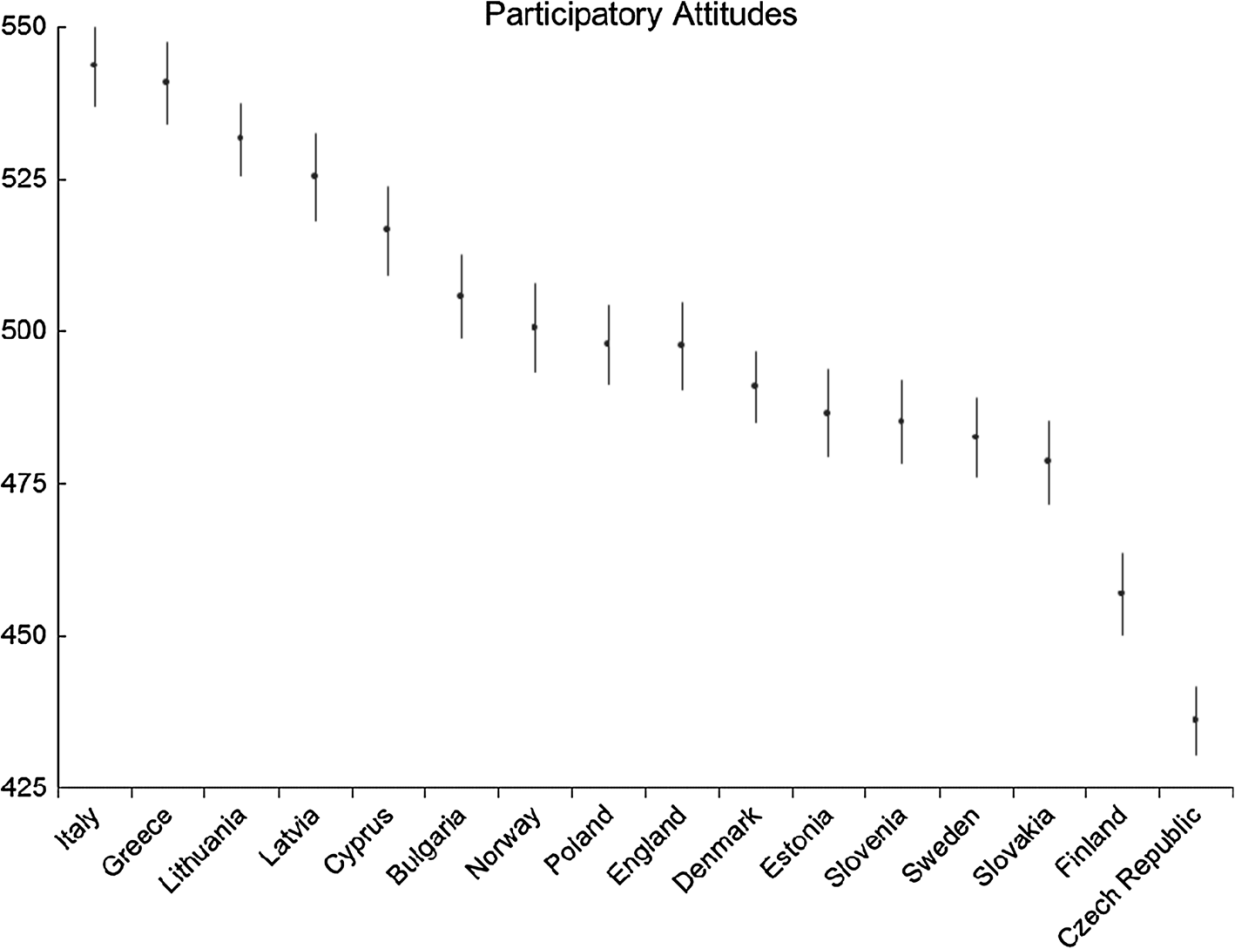
Participatory attitudes: average country scores (with confidence intervals). *Note* confidence intervals are calculated at 95 % level based on a multi-comparison test

**Fig. 7:**
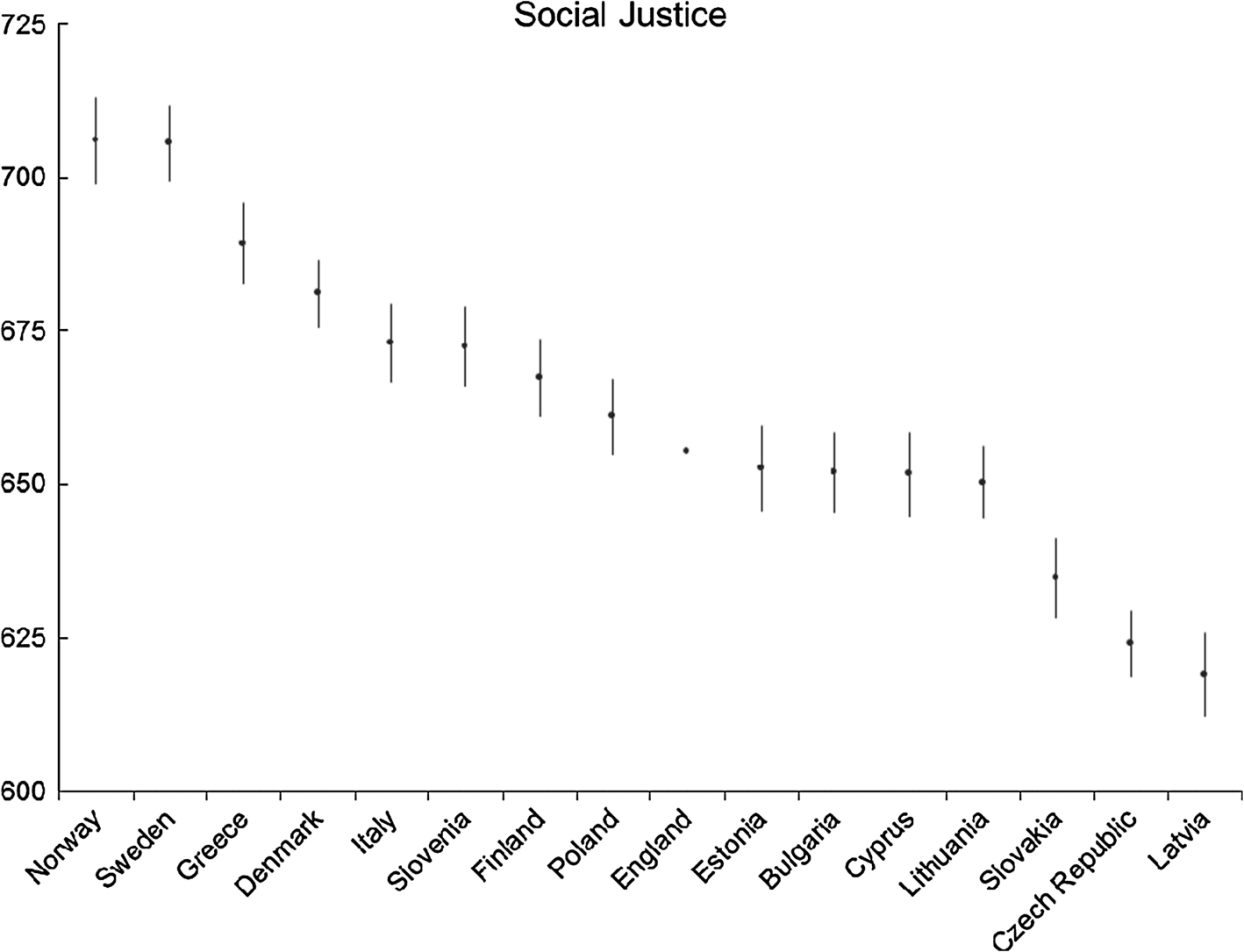
Social justice: average country scores (with confidence intervals). *Note* confidence intervals are calculated at 95 % level based on a multi-comparison test

**Fig. 8:**
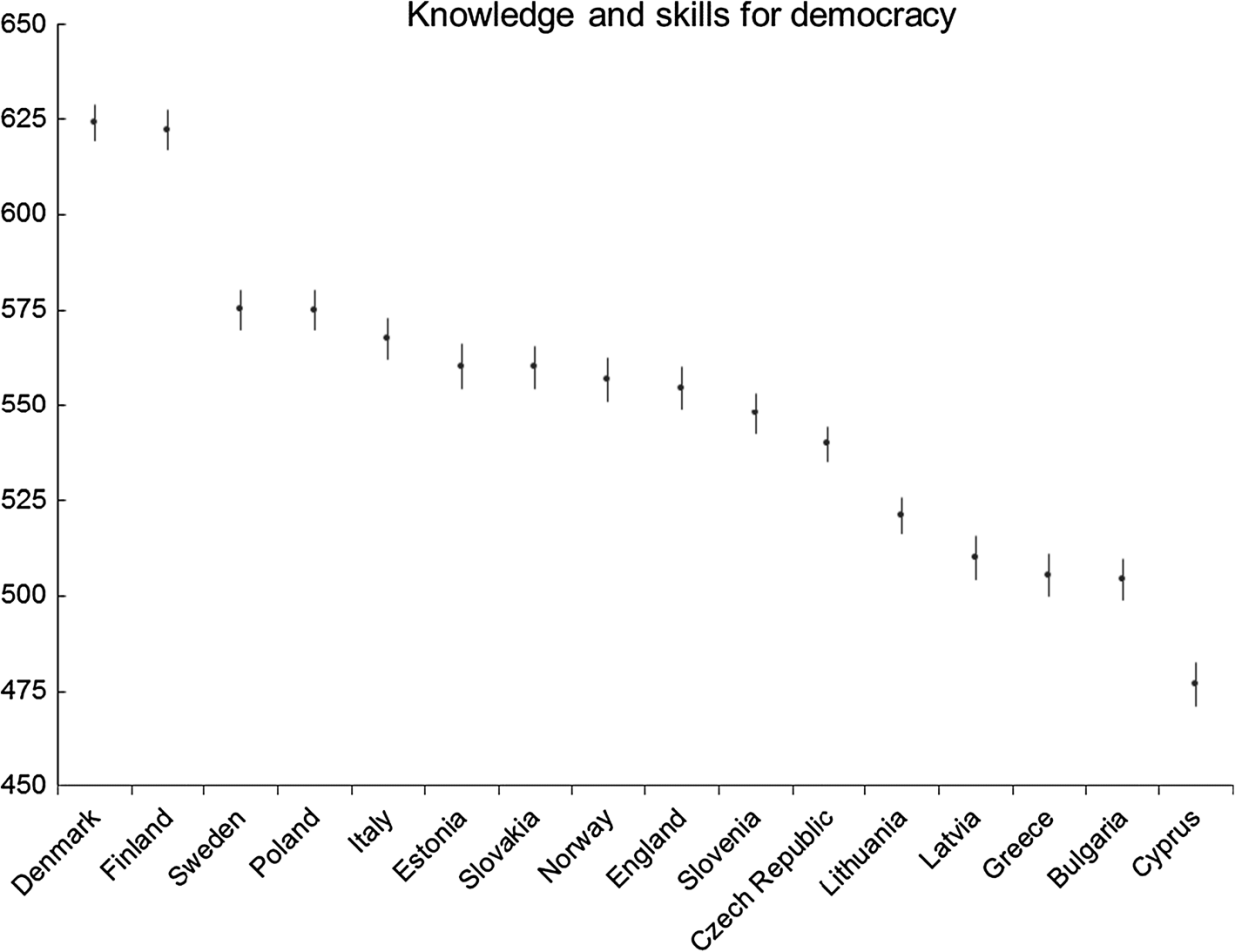
Knowledge and skills for democracy: average country scores (with confidence intervals). *Note* confidence intervals are calculated at 95 % level based on a multi-comparison test

This section begins with results for the overall composite indicator for the 16 countries, followed by the results for the four dimensions of the composite. Country differences across the single composite and the four dimensions of civic competence have been compared using a multiple comparison test (based on information from a balanced, one-way analysis of variance) that compares country means simultaneously, not just in pairs (Searle et al. 1980; Hochberg and Tamhane 1987; Goldstein and Healy 1995). These results are presented as plots in Figs. 4, 5, 6, 7, 8; for each country a confidence interval around its average score was calculated. By checking the overlap of the confidence intervals, one can evaluate statistical significance (here done at the 95 % level). If the intervals overlap, the difference is not significant; if there is no overlap between the intervals the average country scores differ significantly.

### The Civic Competence Composite Indicator

The overall composite indicator results are interesting from the point of countries’ similarities rather than differences (Fig. 4). Despite the variety of experiences of political governance, democracy and citizenship models, in 11 countries the levels of civic competence are relatively similar. Young people of about 14 years old in Italy have the highest levels of civic competence of all the European countries studied. Norway and Greece scored higher than the group of 11 countries, while respondents in the Czech Republic have significantly lower levels of civic competence than all the others. It may not be surprising to find that the Czech Republic, a former communist country, has lower levels of civic competence than other countries in Europe and it could be argued that the civic republican traditions in Italy and Greece facilitate the learning of civic competence there. However, the reasons why countries perform differently are complex and there can be different explanations for different dimensions within the composite. Therefore, the next step is to look into the results for the four dimensions.

### Citizenship Values

The highest scores for ‘citizenship values’ were obtained by Italy and Cyprus (Fig. 5). The next group of countries is Greece, Norway and Bulgaria, followed by Poland, Bulgaria, Lithuania and Latvia, and then by England, Luxembourg, Slovenia. There are three groups of countries with lower levels of norms of citizenship values. The first is Estonia, Sweden, Slovakia, the next the Czech Republic, and finally Finland and Denmark.

The high scores for citizenship values from Italy, Greece, Bulgaria, Poland, Lithuania and Latvia and low from the more stable democracies of Finland and Denmark suggest that the most plausible hypothesis is that young people place greater emphasis on citizenship values in countries with relatively recent transitions to democracy and less experience of democratic stability. The explanation could be that the living memory of the experience of other non-democratic regimes is passed down to younger generations, who then value democracy and democratic engagement more highly. In addition, building on the ideas of Almond and Verba (1963), concepts of civic duties may be idealized rather than based on the substantial experience of democracy on the part of the older generation; it could be the ideal rather than the experience that has been inherited by the young.

In addition, there may be an influence from a civic republican sense of civic duty (Italy, Cyprus and Greece) or even ethnic nationalist discourse, contributing to the scores from former communist countries. In Europe, ethnic as well as civic conceptions of nationalism also have a significant history and tradition, in particular in many of the former communist countries and Germany (Kohn 2008). In the former communist countries, this concept resurfaced after the fall of communism as part of the process of nation-building, with certain sectors of the population glorifying the pre-communist period (Daun and Sapatoru 2008). In this way, those in more recently formed states from the former communist countries tend to have a stronger ethnic concept of citizenship and higher levels of ethnic nationalism, suggesting that in countries such as Latvia, Lithuania, Czech Republic, Slovak Republic and Slovenia people are expected to have higher levels of ‘civic’ duty.

However, Norway is clearly an exception to this theory as it is in the group of highest-scoring countries in Europe, despite having a very different civic culture and political history. Norway, like its Nordic neighbours, is influenced less by ethnic concepts of citizenship and more by both liberal and civic republican traditions than are former communist countries (Telhaug et al. 2006). The liberal values of freedom to engage or not would be expected to weaken concepts of ‘good citizenship’ and civic duty. Moreover, Norway has a long history of stable democracy, despite being invaded and occupied during the Second World War (Hjerm 1998), indicating a realistic experience of democracy within the wider population.

To provide plausible explanations for the higher than expected results from Norway compared to countries with similar socio-political histories, it is helpful to examine citizenship education provision using the data gathered from the latest Eurydice study on citizenship education (Eurydice 2012). Norway, in common with all the Nordic countries, has a long tradition of citizenship education and, according to Eurydice (2012), this has seen continual adaptation based on internal school evaluation and external evaluation of the citizenship education teaching and learning experience, and the school climate (Eurydice 2012). Norway has combined cross-curricular citizenship education aimed at social and cultural competences, along with individual taught lessons in upper secondary education. Class representatives and student councils were made mandatory under the 1998 Education Act and representatives given a consultative role on the majority of decisions taken by school governing bodies (Eurydice 2012). The curriculum also states the need for students to be given an opportunity to experience participation, both in their school and their local community. The results of the ICCS study show that in Norway 95 % of students aged 14 had participated in school elections and 90 %t had participated in multicultural and intercultural activities in the community. According to Eurydice (2012), this is significantly higher than any other country. Since the research suggests that social and civic participation inside and outside school plays a crucial role in enhancing civic competences (Kahne and Sporte 2008; Verba et al. 1995; Campbell 2006; Torney-Purta 2002), one could posit that the high participation rates in both social and civic activities could well be factors that facilitate the affording of importance to citizenship values. However, this does not help to explain why other Nordic countries with a tradition of democratic education, such as Denmark, have significantly lower levels of citizenship values.

### Participatory Attitudes

For ‘participatory attitudes’ (Fig. 6), the results are similar to those of ‘citizenship values’. The highest levels were recorded in a group of countries that includes Italy, Greece, Lithuania, Latvia, and Cyprus, all with similar performance. Next follows a large number of countries, from Bulgaria to Slovakia; three of the Nordic countries; England; Luxembourg; and some former communist countries. The lower end is represented by two countries, Finland and the Czech Republic, the latter having significantly lower levels of participatory attitudes than Finland.

As with the previous dimension of civic values, the highest scores on participatory attitudes are achieved in countries with more recent transitions to democracy. Therefore a similar explanation could be made that the direct experience of the population of non-democratic regimes and fighting for democracy has been passed to the next generation, especially in terms of the ideal of active participation in democracy.

We might have expected, based on existing literature, that countries with strong traditions in democratic schooling and participatory methods, such as the Nordic countries, would have higher levels of participatory attitudes. However, this is not the case. An explanation could be that the high results in Italy are the result of the high proportion, almost 80 %, of teachers who identified ‘promoting knowledge of citizens’ rights and responsibilities’ as one of the most important aims of citizenship education, higher than any other EU country in the ICCS teacher study (Eurydice 2012). In contrast, teachers of the Czech Republic and Finland were below the European average in identifying this priority. Offering this more civic republican style of citizenship education, emphasizing responsibilities as well as rights, might be an additional factor that enhances students’ citizenship values and participatory attitudes.

### Social Justice

The results for the social justice dimension are quite different from those of the dimensions of participatory attitudes, and citizenship values. The European countries that show the highest performances are Norway and Sweden (Fig. 7). These countries are followed by a fairly large group from Greece to Cyprus, which includes most of the countries selected. Next is the Slovak Republic, on its own. Two European countries represent the lowest scores: the Czech Republic and Latvia.

The length of democracy appears to have a positive influence on social justice, as countries with the stable democratic traditions of the Nordic area perform well. These countries have also given greater policy emphasis to cosmopolitan citizenship, including human rights and diversity, which may account for these responses from young people (Telhaug et al. 2006). In contrast, the recent and less stable democracies are found more at the lower end of the table, including Slovakia, Czech Republic and Latvia. All these countries are recently formed nations and their sense of national identity has been to a large extent based upon a common ethnic cultural heritage (Kohn 2008). This can, at least partly, explain the low scores on attitudes towards migrants and minorities. After 1989 ethnic nationalism was used to consolidate power, particularly in newly formed countries like Latvia and Estonia that had experienced high levels of Russian immigration when annexed to the former USSR (Linz and Stepan 2011). In this context, citizenship education became the tool to promote patriotism and loyalty (Toots and Idnurm 2011). Toots and Idnurm (2011) explain that, following the civic republican tradition, ethnic/linguistic minorities even in more recent years have been expected to adopt the culture of the dominant group. Daun and Sapatoru (2008: 157) argue that, for many of the former communist countries in Eastern Europe, in the transition from communism to democracy the notion of equality ‘lost the importance it had enjoyed before 1989’; the emphasis in the education system is now a two-way push to create human capital and enhance national/ethnic identity.

It is helpful to examine more closely the different education offered within these countries. Examining the Nordic education system may also provide an explanation for why these countries perform so well on questions of social justice. After the Second World War, the Nordic countries put equality at the heart of the comprehensive education system they created to remove class differences and enhance the values of ‘equal opportunity, cooperation, adaptation and solidarity’ (Telhaug et al. 2006: 253). As Telhaug et al. (2006) describe, schools were created as microcosms of society where students would meet people from all backgrounds and together participate in the democratic running of the school. Although neoliberalism has recently influenced these Nordic ideals (Wiborg 2010), there is still evidence of these beliefs. For example, the Eurydice report (2012: 61) explains that, ‘[I]n Sweden, both the Education Act as well as the national curriculum state that schools must operate democratically and be a place where both staff and students are empowered to participate in schoolwork and the learning/teaching environment’. The Swedish National Curriculum also requires that parents and teachers actively work together to develop the content and activities of the school (SKOLFS 2010: 18).

However, there are differences in Nordic approaches to citizenship education. In Sweden and Denmark a cross-curricular approach is favoured, whilst in Norway and Finland there are also specific lessons. In Sweden and Denmark, in contrast to Norway, there is little in the way of regulation on how citizenship should be learnt in schools (Eurydice 2012). Thus we could posit that, rather than regulations or specific subjects being the crucial factor for education on social justice, it is more likely that the history and prominence of democracy and equality within the whole education system and society at large plays a role in facilitating social justice values.

Similarly, we can explore the citizenship education of the newer democracies of Latvia and the Czech Republic that gain lower scores on social justice. In their transitions to democracy, former communist countries in Europe all confronted the challenge of changing their education systems, from developing good communist citizens to developing democratic citizens (Buk-Berge 2006). In this regard, the Czech Republic and Latvia have been highly active in recent years constructing citizenship education within the school curriculum, partly as a result of the European Union adopting civic competence as one of its eight key competences (Education Council 2006). Both countries use two approaches to citizenship education: integrated (into a subject called ‘Man and Society’) and a cross-curricular approach. Furthermore, the Latvian school programme also suggests that once a week students should discuss a series of different issues including patriotism (Eurydice 2012: 22) which, depending on the discussion content, may not be conducive to tolerance of minorities or immigrants. Interestingly, the Czech Republic and Latvia share the citizenship curriculum content of ‘Property Ownership’ and ‘Money and the Market Economy’ (Eurydice 2012), which suggests an orientation towards western liberal market ideals of competition rather than any focus on democratic values, tolerance and equality. We could suggest that, despite citizenship education becoming quite prominent in curricula and in contrast to the Nordic model, there are signs of a nationalistic and a liberal market focus on citizenship education that Daun and Sapatoru (2008) describe as the focus for former communist countries in their new education programmes.

### Knowledge and Skills for Democracy

As with the social justice dimension, Nordic countries scored highest in the citizenship assessment, with Denmark and Finland attaining similar scores (Fig. 8). Sweden and Poland form the next group of countries, followed by a large group of countries from Italy to the Czech Republic that are not significantly different. Lithuania can be found next, followed by Latvia, Greece and Bulgaria, also not significantly different. Cyprus forms a group on its own with the lowest score. In general, again we can posit that it is the less wealthy and newer democracies that are the lower performers.

There is little surprise that Finland achieves high scores on cognitive tests, as their pupils typically do well in international assessments such as PISA. At a country level there is a significant and reasonably high correlation (.84) between the cognitive scores for countries that participated in the IEA ICCS study and in the OECD PISA test results. Although this relationship is not known for individual students, the high correlation at the country level may be partly due to the similarity in the cognitive processes tested, for example the capacity to analyse, reason, reflect and evaluate on a written text. In addition, those students with higher reading literacy are more likely to have learnt knowledge on citizenship through reading (Hoskins 2011). Denmark and Sweden, however, perform better on the ICCS citizenship assessment than their students’ performance on the PISA tests. It could be that the Nordic comprehensive and democratic model of education enhances cognitive achievement on citizenship related topics. Poland, however, also performs well on knowledge and skills for democracy compared to other former communist countries, which has been explained by the high level of knowledge of democracy within the population prior to transition (Tobin 2010). Tobin argues that knowledge of democracy had already been cultivated by the active labour unions and by closer involvement and relationships with western democracies long before the collapse of communism. He also highlights how this knowledge enabled teachers to be better prepared to work with the new democratic citizen curriculum.

What is striking is that the countries that perform very well on the citizenship knowledge and skills test are those that had significantly lower scores on citizenship values (Finland and Denmark) and also participatory attitudes (in the case of Finland). One explanation could be that teachers from the top three performing countries—Finland, Denmark and Sweden—all gave high priority to ‘promoting students’ critical and independent thinking’, with at least 80 % of teachers selecting this as a main aim of citizenship education (Eurydice 2012). Perhaps the liberal focus on critical independent thinking that has enhanced Knowledge and Skills on Democracy has also developed critical thought on the concept of the ‘good citizen’, with the unintended effect of underscoring the difficulties of creating real change and reducing enthusiasm for engagement.

## Limitations

The four dimensions of civic competence we have developed and discussed above have been shown to be both conceptually and statistically coherent. Nevertheless, there are several caveats for the indicators that we have developed using the IEA dataset including the age of participants, the breadth in terms of the coverage of civic competence and indicators. These caveats are discussed below.

First, the group represented by the respondents in the study is students aged approximately 14 years old; this study investigates perforce an evolving civic competence in the youngest citizens. Consequently, levels of actual experience of civic engagement, in particular outside the school or family environment, can be expected to be quite limited as young people of this age may, though not necessarily, lack independence for engagement or are restricted from being engaged since they are still minors, or both. According to the national curricula in the participating countries, these students will have experienced some citizenship education, yet the experience of forms of democracy in school tends to increase with age (Eurydice 2012). Despite the clear need to sample older cohorts, however, there is no equivalent European comparative data on civic competence for older age groups that include knowledge, skills, attitudes and values. The IEA CIVED study in 1999 did sample an older cohort of 16-year-old students, but this group was not included in the 2009 study.

The second limitation is the coverage of different aspects of civic competence, which falls beyond our framework due to the availability of data collected in the IEA ICCS study. Although the IEA study provides the most comprehensive quantitative and comparative coverage of youth civic competence to date, it is limited in terms of the breadth of coverage. For example, the study does not include the civic competence civic republican qualities of solidarity (Honohan 2002), nor the critical citizenship qualities of empathy and care (Veugelers 2011) discussed in the literature. Furthermore, as a multiple choice paper and pencil test, the IEA ICCS instruments are not designed to capture key skills such as presenting, persuading and defending ideas that are deemed fundamental to civic engagement. As a result of this limitation of the existing data, our indicator is necessarily restricted to those components of civic competence represented in the dataset.

The third limitation relates to the measurement of any multidimensional phenomenon by means of an index. There are many challenges in monitoring civic competence, from defining the concept itself to rendering it analytically tractable. The added value of a well-constructed index lies in its ability to summarize different aspects of civic competence in a more efficient manner than is possible with a collection of relevant indicators taken separately. Nevertheless, the validity of an index does not merely depend on its statistical soundness but on its acceptance by the community of peers. We propose this CCCI as a useful step to inform research and policymakers, but also as a preliminary step in the ongoing debate on measuring civic competence. Our argument for this suggestion is summarised by Barré (2001), who argues:

quantitative indicators are the starting point for the discussion, with their raison d’être being to be criticized in terms of their (limited) relevance and (limited) comparability. Indicators are considered thus, not as a final result to be accepted, but as an entry point for debate. This is an excellent way to enter an exercise of learning-by-comparing, which is what benchmarking is about. Criticism must be careful and positive, because the purpose is not to dismiss the legitimacy of the exercise, but to help dig further for a better understanding of the situation.

The intention of this research is therefore to provide a tool for monitoring the main dimensions of civic competence in European countries through a learning-by-comparing exercise and to spark further research based on the theories that we have presented. Given the complexity of the concepts described, the discussions and arguments presented need to be considered as hypotheses rather than definitive conclusions.

## Conclusion

The results have shown that the Nordic countries—combining democratically based education systems with teachers who believe that citizenship education is about promoting autonomous critical thinking and a political context of a long and stable democracy—have been able to enhance the social justice and citizenship knowledge and skills of their students (Table 3). However, not all Nordic countries have enjoyed the same success in enhancing young people’ qualities of citizenship values or participatory attitudes, in particular Finland and Denmark. In contrast, the more recent democracies that have stronger nationalistic roots (for example, former communist countries like Latvia and former dictatorships such as Cyprus and Greece) tended to score more highly on citizenship values and participatory attitudes (Table 3). A possible explanation is that these countries have faced much greater political instability in recent years and their youth may well perceive an acute need to engage in either conventional political systems or protest-based activities. At the same time, their parents and the wider community around these student populations have had less exposure to everyday political engagement in the democratic process, limiting the experience that young people can draw upon, suggesting that an ideal picture of good citizenship could be emerging in these countries. Despite changes made to the composite indicator and the survey itself, these findings are similar to the pilot CCCI (Author) constructed using IEA CIVED data from 1999.

**Table 3:** Key findings of this paper

Theories that explain cross national variation	Key findings of this paper
Years of democracy	The years of democracy that a country has experienced (see van Deth et al. 2007 for a related research on adult population) has a positive relationship with young people’s ‘social justice values’ and ‘Knowledge and skills for democracy’
	In contrast, the more recent democracies that have stronger nationalistic roots tended to score more highly on ‘citizenship values’ and ‘participatory attitudes’ for the 14 years old population (a similar to the previous composite, see Hoskins et al. 2011). A possible explanation is that these countries have faced much greater instability in recent years in their democratic system and may well see the acute need to engage either in the conventional political system or through protest-based activities
Influence of concepts of citizenship	Civic republicanism traditions (Lovett 2010) found in Greece and Italy could provide an explanation for their 14 year olds high scores in ‘citizenship values’ and ‘participatory attitudes’ Recently formed nations such as Latvia that have their sense of national identity based upon a common ethnic cultural heritage (Kohn 2008) and where patriotism is still forms part of citizenship education can partly explain the low scores on young people’s attitudes towards migrants and minorities in the ‘social justice’ dimension
	Cosmopolitan policies (Held 2010) related positively to the ‘social justice’ and ‘Knowledge and Skills’ dimensions but the evidence suggests that these principles may well undermine ‘citizenship values’ and ‘participatory attitudes’ in the youth population
Citizenship education	The combination of opportunities for participation and decision making combined with taught courses on citizenship could well be factors that facilitate the qualities of valuing citizenship engagement
	Rather than specific regulations or specific subjects being the crucial factor for citizenship education it is more likely that the history and prominence of democracy and equality within the whole education system and society at large plays a role in facilitating social justice values in the youth population
	Offering more civic republican style to the citizenship education that emphasizes responsibilities as well as rights could well be an additional factor that enhances concepts of the good citizens
	Perhaps the focus on critical independent thinking has enhanced knowledge and skills for democracy but has also developed critical thoughts on the concept of the ‘good citizen’, which may have the unintended effect of underscoring the difficulties of creating real change, thus reducing their enthusiasm for engagement

It is interesting to reflect on the distinctions between the four dimensions. The correlations performed at the individual level showed a relationship between Knowledge and Skills for Democracy, and social justice values, but no relationship between Knowledge and Skills and either citizenship values or participatory attitudes. The highest correlation was between citizenship values and participatory attitudes. Combining the individual level results with country-level information on education strategies, we could suggest that the Nordic model of education enhances more of the qualities of both knowledge and skills for democracy, and social justice values and, in contrast, the newer democracies that include a civic or ethnic nationalistic element within citizenship education enhance the qualities of both participatory attitudes and citizenship values. There are few countries that facilitate the learning of all four dimensions of civic competence, which suggests that different strategies facilitate the learning of particular dimensions. It is possible that the Nordic teachers’ priority for developing liberal critical-autonomous citizens facilitates cognition of citizenship and equality values, but may be a less fruitful approach to enhance participatory attitudes or concepts of a ‘good’ citizen. These may be better supported by Italian teachers’ prioritization of responsibility, drawing on civic republican traditions. Further research is therefore necessary at the individual level, on the relationship between the different dimensions of civic competence and how they are learnt. An examination of the role of national culture may also be relevant here.

Finally, it should be remembered that we do not have full knowledge of the relationship between a 14-year-old’s civic competence and how this changes during the transition into adult civic life. Tentative research findings based on cross-sectional survey data have shown that the comparative lack of enthusiasm for participation in Nordic 14-year olds is not found a few years later (Amnå and Zetterberg 2010). Amnå and Zetterberg compare the rates of intended participation in Nordic youths and their southern European counterparts in the CIVED database, and then the adult European Social Survey dataset, they note that by age 17–25 the enthusiasm in Southern Europe has already gone and, in fact, interest, voting and protesting is much higher among Nordic youth. The authors note that the level of intended participation by 14-year olds in the Nordic countries is actually more realistic than that of the Southern European cohort, and suggest that the reason for the differing processes of democratic transitions across regions is that young people in the Nordic countries have greater opportunities than their Southern European counterparts for political engagement as they become older. Youngsters in Nordic countries are encouraged to engage in a wide range of public activities in the formative period of their late teens and this encourages authentic involvement by a reluctant youth, whereas young Southern European adolescents’ intention to participate merely dissipates.

Future research and surveys in the field of civic competence need also to focus on regular monitoring of older cohorts (roughly aged 16 years), and on the measurement of the civic republican qualities of solidarity, critical citizenship qualities of empathy, and care and skills related to presenting, persuading and defending ideas deemed fundamental to civic engagement. Finally, in order to compare the different patterns in youth democratic transitions between countries in Europe over time, further research should be both comparative and longitudinal.
